# Global advances and challenges in perinatal psychiatry

**DOI:** 10.1192/j.eurpsy.2025.262

**Published:** 2025-08-26

**Authors:** N. Jovanovic

**Affiliations:** Queen Mary, University of London, London, United Kingdom

## Abstract

**Abstract:**

Perinatal psychiatry is a rapidly evolving subspecialty focused on diagnosing, recognising early, and treating mental disorders in pregnant and postpartum individuals, up to two years after childbirth (timeframes vary globally). The field has gained attention as governments around the world work to meet the United Nations Sustainable Development Goals (SDGs), particularly SDG 3 (health and well-being) and SDG 5 (gender equality), aiming to improve maternal, infant, and child health outcomes.

In most countries, perinatal mental illness is managed by family doctors, midwives, and general adult psychiatrists, although some countries have developed specialist community and inpatient perinatal mental health services. The goal is effective treatment to improve pregnancy outcomes, parental mental health, social functioning, and child development. Studies show that infants of mothers with untreated perinatal depression and psychosis are at higher risk of long-term mental health disorders and poor social and educational outcomes. Early detection and efffective and acceptable interventions are key to breaking this cycle.

This presentation will address global advances and ongoing challenges. Significant progress includes increased awareness, emerging specialist services, novel pharmacological treatments with improved safety profiles, expanded evidence-based psychosocial interventions, and a focus on maternal suicide prevention, a key driver of maternal mortality. However, major challenges remain: perinatal suicide is a leading cause of maternal death, poor quality of evidence for prescribing in pregnancy and lactation, long-term morbidity due to untreated mental illness is often overlooked, and access to care is limited especially in low- and middle-income countries. Social stigma and structural barriers also prevent many from seeking help. Addressing these issues requires a global, multidisciplinary approach, integrating public health initiatives, policy changes, workforce training, and research innovations to ensure equitable access to high-quality perinatal mental health care worldwide.

**Disclosure of Interest:**

None Declared

